# Enlightening the association between *TNF*-α -308 G > A and migraine: a meta-analysis with meta-regression and trial sequential analysis

**DOI:** 10.1186/s12883-023-03174-x

**Published:** 2023-04-21

**Authors:** Amrit Sudershan, Srishty Sudershan, Mohd Younis, Meenakshi Bhagat, Agar Chander Pushap, Hardeep Kumar, Parvinder Kumar

**Affiliations:** 1 Institute of Human Genetics, University of Jammu, Jammu & Kashmir, Jammu, India 180006; 2 Department of Human Genetics, Sri Pratap College, Cluster University of Srinagar, Jammu & Kashmir, Kashmir, India; 3 Department of Zoology, University of Jammu, Jammu and Kashmir, Jammu, India 180006; 4 Department of Education, Dakshina Bharat Hindi Prachar Sabha, Madras, India 600017; 5 Department of Neurology, Super Specialty Hospital, Jammu and Kashmir, Jammu, India 180006

**Keywords:** Migraine, *TNF-α*, rs1800629, Migraine, *-308 g* > *a*

## Abstract

**Background:**

Migraine is a complex neurological disorder that is characterized by a "lower threshold of neuronal hyperexcitability" with distinctive periodicity and complex vascular dysfunction. Genetic factors have impacted incredibly on the susceptibility of migraine and one such example is the *TNF-α* 308G > A.

**Aim:**

Therefore, we aim to provide a glimpse of the association of the *TNF-α* 308G > A risk on the susceptibility of migraine.

**Method:**

The pooled odds ratio with the associated 95% of confidence interval were calculated using different genetic models. Heterogeneity was accessed by using Cochran's Q Test and I^2^ statistics and Begg's and Egger's tests were used for finding the publication bias, tests were two-sided, and a *p*-value of < 0.05 was considered statistically significant. The Trial Sequential Analysis with Meta-regression Analysis were also utilized to find out the sample size requirement for meta-analysis to avoid type I error and source of heterogeneity respectively.

**Result:**

A total of 13 studies with cases: 7193 and controls: 23,091 were included and after using different genetic models, no overall association with migraine and its clinical subtype migraine with aura was observed (Allele model “OR: 1.28, 95% C.I. [0.96–1.69] and OR: 0.99,95% C.I. [0.69–1.42]) respectively. Interestingly, after sub-grouping using the “ethnicity criteria” in the migraine group, it was observed that the allelic genetic model and the dominant model were found to be significantly associated with the Asian ethnic group (OR: 1.79, 95% C.I. [1.13–2.84], and OR: 1.85, 95% C.I. [1.0927; 3.1580].

**Conclusion:**

In conclusion, the present meta-analysis has provided evidence that 308G > A increases the risk of migraine only in the Asian population.

## Introduction

Lower neuronal hyperexcitability is used to define migraine, which is a complex neurological disorder characterized by distinctive periodicity and complex vascular dysfunction [1]. According to the International Classification of Headache Disorders (ICHD-3), migraine is classified into episodic migraine which is further sub-classified into Migraine without Aura (MO), Migraine with Aura (MA), and Chronic Migraine (CM). The overall prevalence rate of the diseases is roughly 21.7% with a 12–15% average variance between nations [2–4] where it impacted every age group, including younger children (2.7 to 10.0%), where both sexes are equally affected, while adult females (12–17%) are more likely to experience them than males (4–7%) [5, 6].

Neurogenic neuro-inflammation, defined as "inflammatory reactions in central and peripheral parts of the trigeminovascular system in response to neuronal activity," has piqued the interest of migraine researchers in recent years [7] due to its critical role in the processing, integration, and transmission of sensory information in migraine pathogenesis [1, 8, 9]. *TNF-α* is an astonishing example of a proinflammatory cytokine involved in inflammation initiation and is secreted by the microglial cell upon activation [10].

**Fig. 1:**
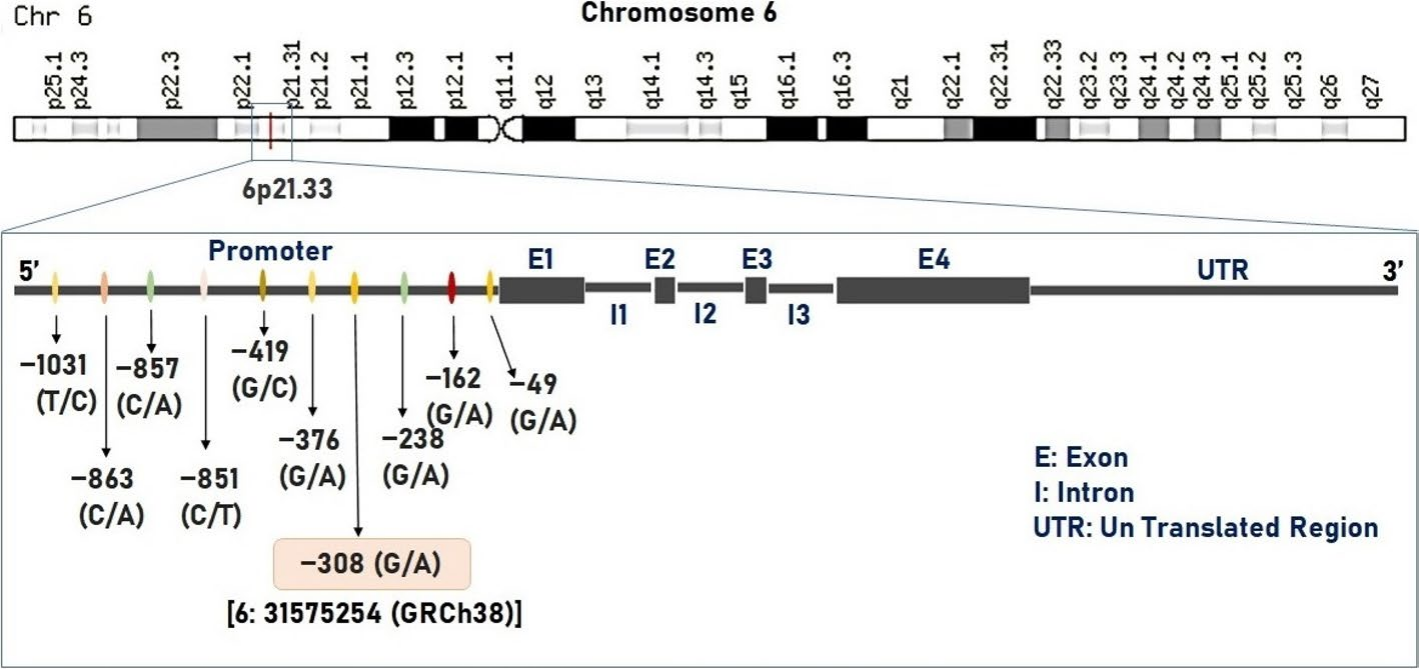
Location and structure of TNF-alpha gene and list of upstream variants

*TNF-α* (NCBI Entrez Gene: 7124) encodes for a cytokine that promotes a diverse range of proinflammatory reactions and is responsible for various conditions including migraine (MalaCards—human disease database and OMIM—Online Mendelian Inheritance in Man). There are diverse numbers of Single Nucleotide Variations (SNVs) presented in the upstream region of *TNF-α* (Fig. 1) [11]. One such variant is located -308 upstream of the gene and is known for its regulatory activity and is named “-308 G > A polymorphism/ rs1800629”. This G to A polymorphism shows different allele frequencies within the different populations (Ensembl.org). Greater baseline/constitutive and inducible TNF-α expression has been linked to the uncommon 308A allele both in-vivo and in-vitro studies [12] and is associated with elevated plasma levels [13]. Multiple researchers have found an increased risk of diseases with the presence of *TNF-α* 308A on a wider scale, across different populations [14–17].

Given that the -308 G > A polymorphism is thought to affect the TNF-α gene's promoter activity and that the gene is part of the major histocompatibility complex, this variant could interfere with immunologic equilibrium and impact migraine genesis, development, or progression. Considering the aforementioned data, numerous case–control and cohort research have been performed, to investigate the association between migraine risk and the -308G/A variant but, the results were found inconsistent. Diversity might be due to the low power of the individual study, diverse ethnic groups, and possible selection bias. Numerous meta-analyses have been published in the past to help overcome the constraints of individual investigations [18–21], but showed the controversial outcome. Therefore, we conducted an updated meta-analysis featured with Trial sequential analysis (TSA) and Meta-Regression Analysis (MRA) in order to enhance statistical power and derive a more precise result of the association between the rs1800629 and the risk of migraine.

## Method

### Literature survey

Using the approach of “systematic way of literature survey” according to the PRISMA (Preferred Reporting Items for Systematics Reviews and Meta-Analysis) guidelines [22] from the online database such as PubMed and research article search engine i.e., Google scholar, potential studies that examine the relationship between -308G/A variant and migraine risk were identified by our three authors (S.S, M.B, & A.C.P). Multiple key terms were used in our search strategy, including “*TNF-α* OR TNF-α gene OR Tumor necrosis factor-alpha gene AND variant OR mutation OR polymorphism OR AND -308 OR rs1800629 AND Migraine disorder”. The search procedure lasted up to May/10/ 2022, and the language restrictions were English. Since PubMed has more than 34 million citations for scientific publications from sources including MEDLINE, life science journals, and e-books, we didn't search any additional databases.

### Inclusion and exclusion features

The following inclusion features have to be satisfied by the studies included in this meta-analysis such as (1) research must have investigated the correlation between the -308G/A and migraine predisposition (2) studies must be cohort designed or a case–control design, (3) research need to yield sufficient information to compute ORs and the related CIs of 95% and also other factors described in (Table 1).

**Table 1:** Inclusion and exclusion criteria

**S. No**	**Inclusion criteria**
**1**	A cross-sectional, case–control, or cohort study design is required
**2**	Authors must investigate patients according to the criteria of the International Headache Society (IHS) and ICHD-3
**3**	The authors must have looked at the genetic polymorphisms found in TNF-alpha
**4**	The genotype frequencies for the polymorphisms studied among migraineurs and non-migraineurs must be reported in the paper
**5**	The largest research with acceptable genetic data was included in publications with overlapping cases and/or controls
**6**	If article does not have the required data the data were taken from a previously published article
**7**	All studies must be within the Hardy–Weinberg Equilibrium

Studies were excluded if they did not meet all criteria

### Data extraction and quality assessment

Different features from each study were extracted including the demographic characteristic including the country name and ethnicity, the numbers of patients and healthy groups, also the cohort data, genotypic frequency from both cases and controls, and first authors with years of publication and lastly what type of technique utilized to determine the genotype? Each published research study's quality was evaluated using the Newcastle–Ottawa quality assessment scale (NOS), and a score of six points was considered to be a good study (Ottawa Hospital Research Institute (ohri.ca).

### Statistical analysis

To find out the strength of an association between the rs1800629 and migraine susceptibility, the pooled Odds Ratio (OR) (OR > 1: the odds of exposure among case are greater than odds of exposure among controls & OR < 1: the odds of exposure among case are lower than the odds of exposure among controls) with associated 95% of Confidence Interval (CI) were calculated. In order to evaluate the Hardy–Weinberg equilibrium (HWE) in the control groups, the Chi-square statistic was used. To find out the impact of the risk factor on disease susceptibility, four different genetic models including allele model (A vs. G), AA vs. GG + GA (recessive model), AA + GA vs. GG (dominant model), and GA vs AA + GG (over-dominant model). To evaluate how each study affected the total OR and 95% CI, a sensitivity analysis was also performed.

Using the Cochran's Q Test and I^2^ statistics as a test for heterogeneity of the research studies included in this meta-analysis, different level of heterogeneity was defined which includes low (up to 25%), moderate (> 25% to 75%), and (above 75%) degrees of heterogeneity. The random effect model was only used when the test of heterogeneity i.e., I^2^ will be above 75%, and notably, the publication bias including reporting bias was assessed using Begg's and Egger's tests. All tests were two-sided, and a *p*-value of < 0.05 was considered statistically significant. Due to easy graphical user interphase (GUI), the Meta-Genyo online Statistical Analysis System software was used for all statistical analyses (MetaGenyo: Meta-Analysis of Genetic Association Studies).

### Meta-Regression analysis

Subgroup analysis and Bayesian meta-regression analysis based on years of population, ethnicity, diagnostic criteria, and genotyping technique were carried out to find specified sources of heterogeneity across included research. Using Microsoft Excel-2019 with Analysis ToolPak, data on parameters like R-square, intercept coefficient, standard error, t-value, confidence interval, and *p*-value were retrieved for the Bayesian meta-regression.

### Trial sequential analysis

A unique approach known as Trial Sequential analysis (TSA) has been employed in the current meta-analysis to limit random mistakes by determining whether the studies contained in the meta-analysis have exceeded the required sample size or not. Because chances of random error increase in the meta-analysis due to the repeated significance tests and continuous data distribution which will ultimately cause the Type I error [23]. TSA tool (Copenhagen Trial Unit, Denmark) was used to calculate the needed information size based on a 5% overall risk and a relative risk reduction of 20% (with 80 percent power) for assessing meta-analysis reliability (TSA – ctu.dk). However, there are two main possibilities: if the cumulative Z value/curve crosses the RIS (Required Information Size), no further studies are required,and if the Z curve does not exceed the RIS threshold, the sample size is insufficient and more reliable studies are required.

## Result

### Study characteristics

**Fig. 2:**
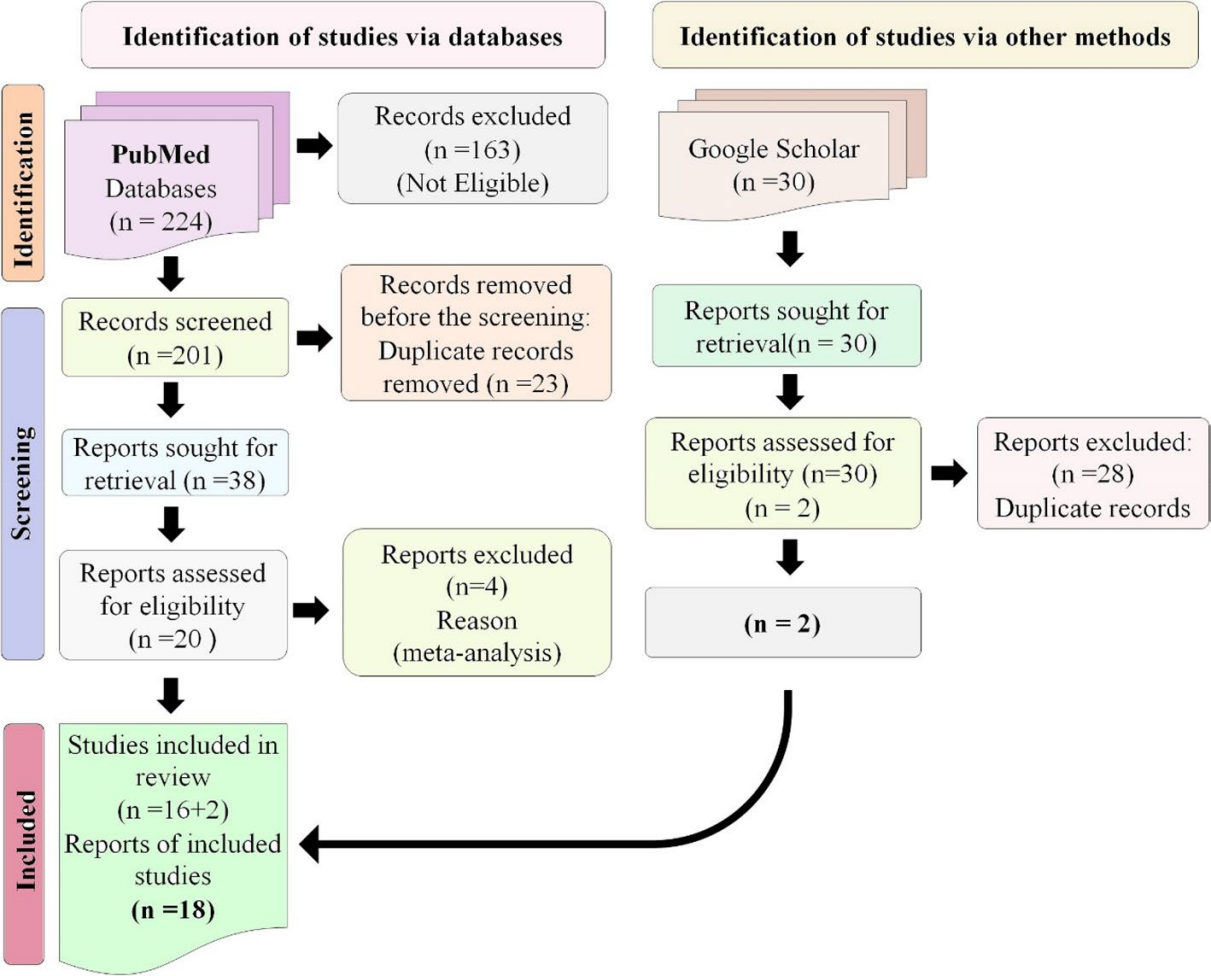
Systematic representation of the PRISMA criteria used in meta-analysis

The studies used for this meta-analysis meet the PRISMA requirements and the selection flow diagram depicted in (Fig. 2). We found a total of 18 studies but after founding one duplicated study [24] only 17 were found to be eligible studies with the pooled case numbers of 7692 and 23,570 controls concerning 308G > A (Table 2) were included in the meta-analysis. But after finding the studies that were not found in HWE [22–25], only 13 studies (cases: 7193 and controls: 23,091) were determined to be suitable for further examination.

**Table 2:** Features of different association study utilizing case–control and cohort study design

**Study name**	**Diagnosis**	**Case/controls**	**Source Of controls**	**Country**	**Ethnicity**	**Genotyping method**	**Case**			**Control**			**NOS**	**HWE**
							**GG**	**GA**	**AA**	**GG**	**GA**	**AA**		
**Trabace et al., 2002 **[25]	HIS	79/101	PB	Italy	Caucasians	PCR–RFLP	67	12	0	90	9	2	7	**0.048**
**Rainero et al., 2004 **[26]	HIS	299/306	HB	Italy	Caucasians	PCR–RFLP	256	42	1	207	88	11	7	*0.858*
**Herken et al., 2005 **[27]	HIS	60/62	HB	Turkey	Asian	PCR–RFLP	54	5	1	53	9	0	6	*0.858*
**Mazaheri et al., 2006 **[28]	HIS	221/183	HB	Iran	Persian	PCR-SSP	51	163	7	94	86	3	6	**0.0128**
**Lee et al., 2007** [29]	HIS	439/382	HB	Korea	Asian	PCR	377	61	1	338	41	3	6	*0.3849*
**Ghosh et al., 2009 **[30]	HIS	216/216	HB	India	Asian	PCR–RFLP	175	41	0	191	24	1	7	*0.858*
**Asuni et al., 2009 **[31]	ICHD-II	299/278	HB	Italy	Caucasians	PCR	272	26	1	249	28	1	7	*0.858*
**Schurks et al., 2009 **[32]	SR	4577/20425	PB	USA	Caucasians	Mass-array	3081	1369	127	13,947	5877	601	6	*0.858*
**Pappa et al., 2010 **[33]	ICHD-II	103/178	HB	Greek	Caucasians	PCR–RFLP	89	14	0	145	31	2	8	*0.858*
**Yılmaz et al., 2010 **[34]	ICHD-II	67/96	HB	Turkey	Asian	PCR–RFLP	37	23	7	79	16	1	6	*0.858*
**Ates et al., 2011 **[35]	HIS	203/202	HB	Turkey	Asian	ARMS-PCR	125	78	0	162	40	0	6	*0.3155*
**Stuart et al., 2013 **[36]	HIS	335/345	HB	Australia	Caucasians	HRM-RFLP	220	95	20	230	97	18	7	*0.292*
**Fawzi et al., 2015 **[37]	HIS	200/200	HB	Egypt	Egyptian	PCR–RFLP	136	51	13	169	29	2	8	*0.858*
**Shaik et al., 2018 **[38]	N/A	129/129	HB	Malaysia	*Asian*	PCR–RFLP	99	30	0	125	4	0	6	*0.858*
**Hamad et al., 2021 **[39]	HIS	183/184	PB	Jordan	Asian	PCR–RFLP	96	72	15	51	108	25	8	**0.048**
**Kesavan et al., 2021 **[21]	HIS	212/218	HB	India	Asian	ARMS-PCR	158	38	16	152	56	10	7	*0.3155*
**Tatlisuluoglu et al., 2021 **[40]	HIS	70/ 65	HB	Turkey	Asian	PCR–RFLP	2	38	30	0	52	13	6	**0**

*SR* Self-Reported, *HIS* International Headache Society, *HWE* Hardy Weinberg Equilibrium, *NOS* Newcastle Ottawa quality Scale, *HB* Hospital Based, *PB* Population Based, *ICHD-II* International Classification of Headache Disorder, *PCR–RFLP* Polymerase Chain Reaction-Restriction Fragment Length Polymorphism, *ARMS-PCR* Amplification-Refractory Mutation System- Polymerase Chain Reaction

**Not in HWE**

*Population in HWE*

### Meta-analysis

**Fig. 3:**
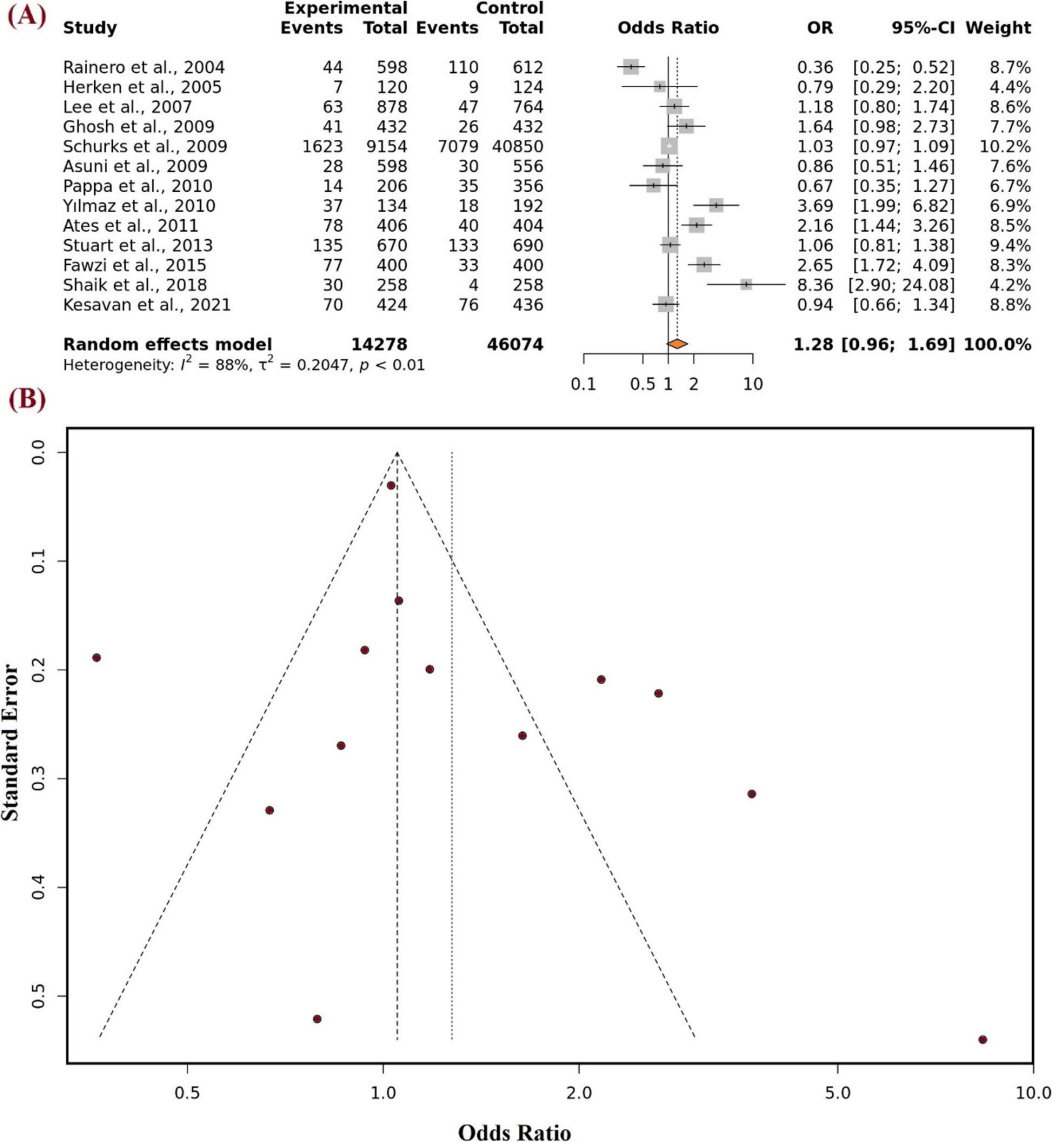
**A** Forest plot representing the Odds ratio (OR) utilizing the 95% of Confidence interval (C.I.) for all the individual studies featured with different sample size and also the pooled odds ratio (OR) with 95% C.I. for Allelic model. **B** Funnel plot of TNF-alpha -308 G > A and susceptibility of migraine in diverse population utilizing Allele model

This currently updated meta-analysis utilizes the different genetic models to observe the effect of the rs1800629 variant of *TNF-α* on migraine susceptibility. In the first model i.e., the allelic model, polled results from the experiment group (*n* = 14,278) and control (*n* = 46,074) showed the non-significant association value of “OR: 1.28, 95% C.I. [0.96–1.69]. The impact of the mutant allele was seen after using the random effect model as the “test of heterogeneity (I^2^) was 88% (Fig. 3A). After adjusting for the dominant model and utilizing the random effect model (I^2^), a non-significant strength of association was found OR: 1.29, 95% C.I. [0.95–1.75]. As the value for the test of heterogeneity was found below the moderate threshold (55%), a fixed effect model was then utilized to check out the effect of the recessive genetic model on migraine susceptibility and found an OR: 1.00, 95% C.I. [0.84–1.19]. For the analysis of the over-dominant genetic model with an I^2^ = 84%, the random effect model showed the associated value OR: 1.22 [0.91–1.64].

**Fig. 4:**
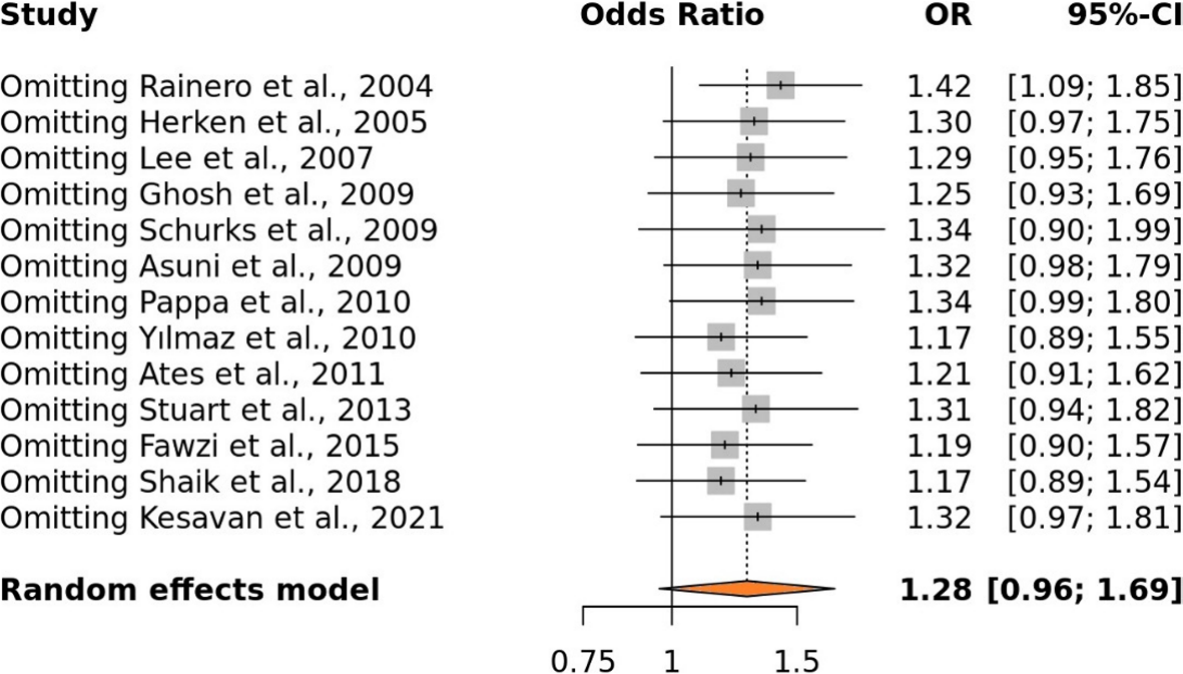
Sensitive analysis representing allele model

Using Egger's test, which is based on the relationship between standard error and strength of association, the publication bias was evaluated. The plotted articles with high accuracy on top and low accuracy down in the plot formed the perfect funnel-shaped structures. Such symmetrical funnel plots were formed for all genetic models which direct that there was no publication bias (Fig. 3B). A sensitive analysis was also performed for all genetic models by removing each research one at a time. It was shown that none of the pooled ORs were significantly impacted, demonstrating the strong stability of the meta-analysis findings (Fig. 4).

After sub-grouping using the criteria of ethnicity for the migraine group, it was observed that the allelic genetic model and the dominant model were found to be significantly associated with the Asian ethnic group (OR: 1.79, 95% C.I. [1.13–2.84], and OR: 1.85, 95% C.I. [1.09; 3.15] (Table 3). Also, it was observed that the recessive genetic model, an over-dominant genetic model was found to increase the risk but did not reach statistical significance. A highly statistically significant association was found in the Egyptian population for all genetic models with recessive showed the highest (OR: 6.88, 95% C.I. [1.53–30.90]) (Table 3).

**Table 3:**
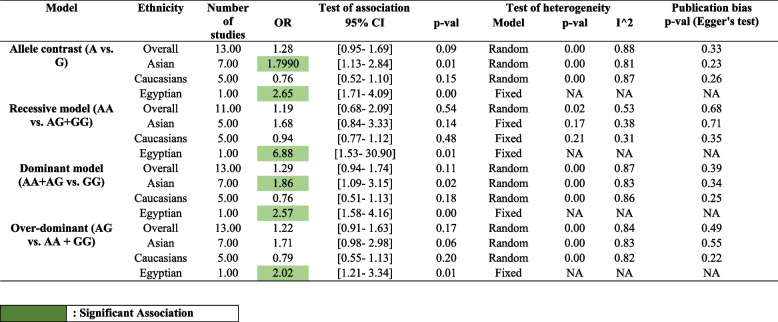
Sub-grouping of the migraine case based on the ethnicity

**Fig. 5:**
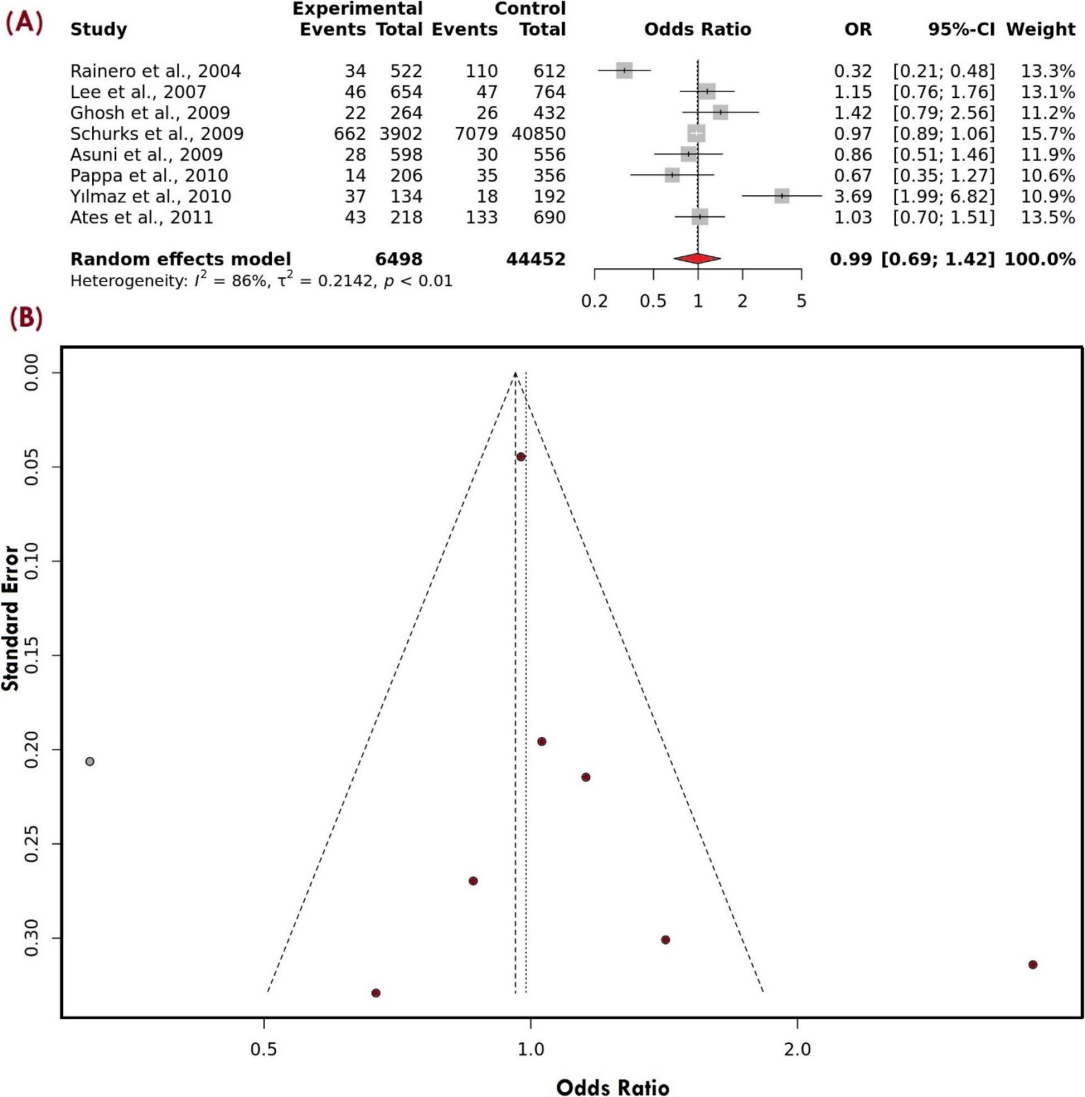
**A** Migraine with aura: Forest plot representing the Odds ratio (OR) utilizing the 95% of Confidence interval (C.I.) for all the individual studies featured with different sample size and also the pooled odds ratio (OR) with 95% C.I. for Allele model. **B** Migraine with aura: Funnel plot of TNF-alpha -308 G > A and susceptibility of migraine in diverse population utilizing Allele model

**Fig. 6:**
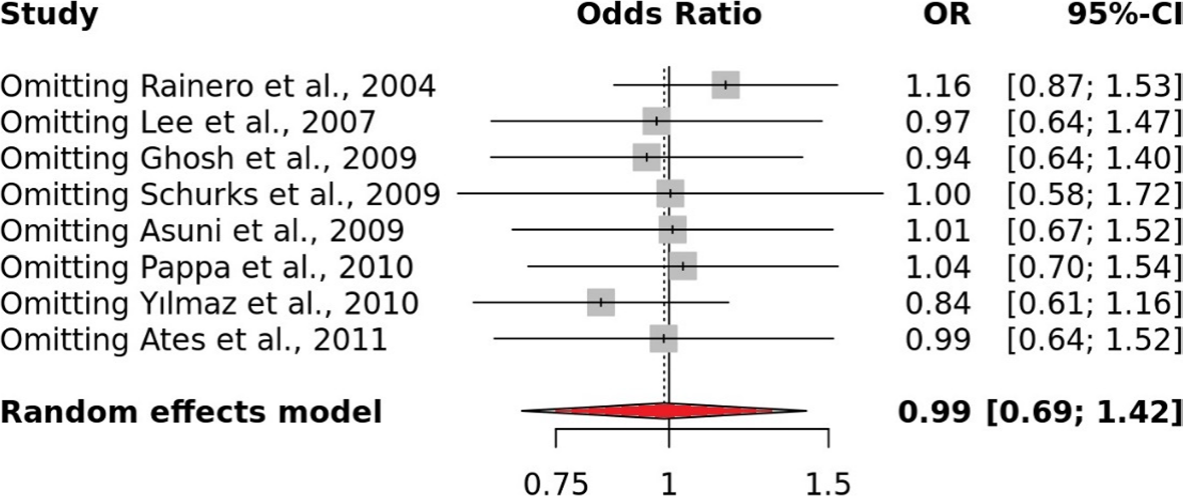
Sensitivity analysis to evaluate the stability of pooled results of migraine with aura Allele model

Apart from the ethnicity sub-grouping, clinical feature subgroup analysis of migraine i.e., migraine with aura was also carried out to see if there is any significant association between the rs1800629 and the susceptibility of MA. We observed that after excluding the studies, with no MA cases/ no inclusion of MA cases and the studies which don’t fall under HWE, a total of 8 studies were found with a total sample size of 25,475 (case = 3249 and control = 22,226) (Table 4). For the allelic model, as the I^2^ was found 86% and after using the random effect model, no association was found OR: 0.99,95% C.I. [0.69–1.42] (Fig. 5A). For the dominant model, the I^2^ was above the high threshold, therefore, after using the random effect model the association value was OR: 1.00, 95% C.I. [0.68–1.45]. The recessive and the over-dominant model show a non-significant association with an association value i.e., OR: 0.96, 95% C.I. [0.74–1.24], and (OR: 0.99, 95% C.I. 0.71–1.38] after adjusting for the fixed effect model (I^2^: 36%) and the random model respectively. Sub-grouping of MA based on the ethnicity criteria revealed no association between rs1800629 and migraines in Asian and Caucasian populations (Table 5). To evaluate the publishing bias, all funnel plots seemed symmetrical, indicating a negligible publication bias (Fig. 5B). Sensitivity analysis also indicated the high stability of the meta-analysis results (Fig. 6).

**Table 4:** MA Sub group analysis based on ethnicity

Study	Ethnicity	Control	Teq	GG case	GA case	AA case	GG control	GA control	AA control
Rainero et al., 2004 [26]	Caucasians	HB	PCR–RFLP	228	32	1	207	88	11
Lee et al., 2007 [29]	Asian	HB	PCR	282	44	1	338	41	3
Ghosh et al., 2009 [30]	Asian	HB	PCR–RFLP	110	22	0	191	24	1
Schurks et al., 2009 [32]	Asian	PB	ARMS-PCR	1346	548	57	13,947	5877	601
Asuni et al., 2009 [31]	Caucasians	HB	PCR–RFLP	272	26	1	249	28	1
Pappa et al., 2010 [33]	Caucasians	HB	PCR	89	14	0	145	31	2
Yılmaz et al., 2010 [34]	Caucasians	HB	PCR–RFLP	37	23	7	79	16	1
Ates et al., 2011 [35]	Asian	HB	PCR–RFLP	71	33	5	230	97	18

**Table 5:** MA Sub group analysis based on ethnicity

**Model**	**Ethnicity**	**Number of studies**	**Test of association**			**Test of heterogeneity**			**Publication bias** ***p*-value (Egger's test)**
			**OR**	**95% CI**	***p*-value**	**Model**	***p*-value**	**I**^**2**^	
**Allele contrast (A vs. G)**	Overall	8	0.98	[0.68- 1.41]	0.94	Random	0	0.863	0.9694
	Asian	4	0.99	[0.91- 1.07]	0.82	Fixed	0.5506	0	0.1
	Caucasians	4	0.89	[0.32- 2.47]	0.82	Random	0	0.9306	0.2333
**Recessive model (AA vs. AG + GG)**	Overall	8	0.96	[0.74- 1.24]	0.76	Fixed	0.1423	0.3588	0.5854
	Asian	4	0.96	[0.74- 1.25]	0.80	Fixed	0.844	0	0.0944
	Caucasians	4	0.8	[0.08- 7.54]	0.84	Random	0.0183	0.701	0.9122
**Dominant model (AA + AG vs. GG)**	Overall	8	0.99	[0.68- 1.45]	0.98	Random	0	0.8467	0.9089
	Asian	4	0.99	[0.90- 1.09]	0.88	Fixed	0.379	0.0269	0.0761
	Caucasians	4	0.88	[0.32- 2.41]	0.81	Random	0	0.9169	0.143
**Over-dominant (AG vs. AA + GG)**	Overall	8	0.99	[0.71- 1.37]	0.96	Random	0	0.7852	0.8792
	Asian	4	0.99	[0.90- 1.09]	0.96	Fixed	0.2607	0.2513	0.06
	Caucasians	4	0.84	[0.37- 1.89]	0.67	Random	0.0001	0.8658	0.1054

### Trial sequential analysis

**Fig. 7:**
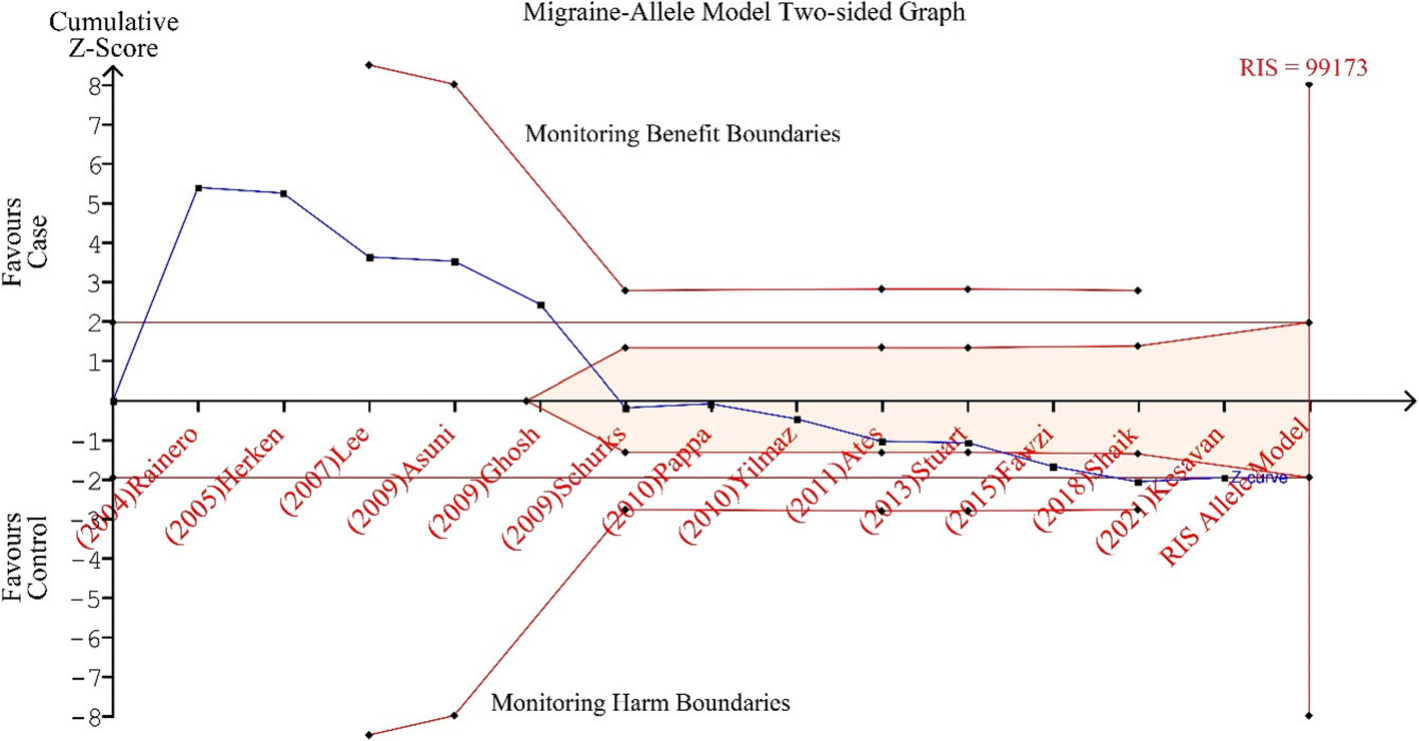
Migraine (Allele Model): Trial sequential analysis (TSA) of the studies included in the meta-analysis of TNF-alpha 308 G > A polymorphism allele model based on a 5% overall risk of false positive error and a relative risk reduction of 20% (with 80 percent power). The cumulative Z-score did not cross the TSA monitoring threshold and the RIS line therefore indicates more studies are needed to find out the precise association

The necessary sample size and the reliability of the meta-analysis were calculated using TSA statistics and it was noted that the cumulative Z score did not cross the Required Information Size for allele models in migraine (Fig. 7). Therefore, the TSA has shown that more sample size will still be needed to find out the precise association.

### Bayesian meta-regression analysis

**Fig. 8:**
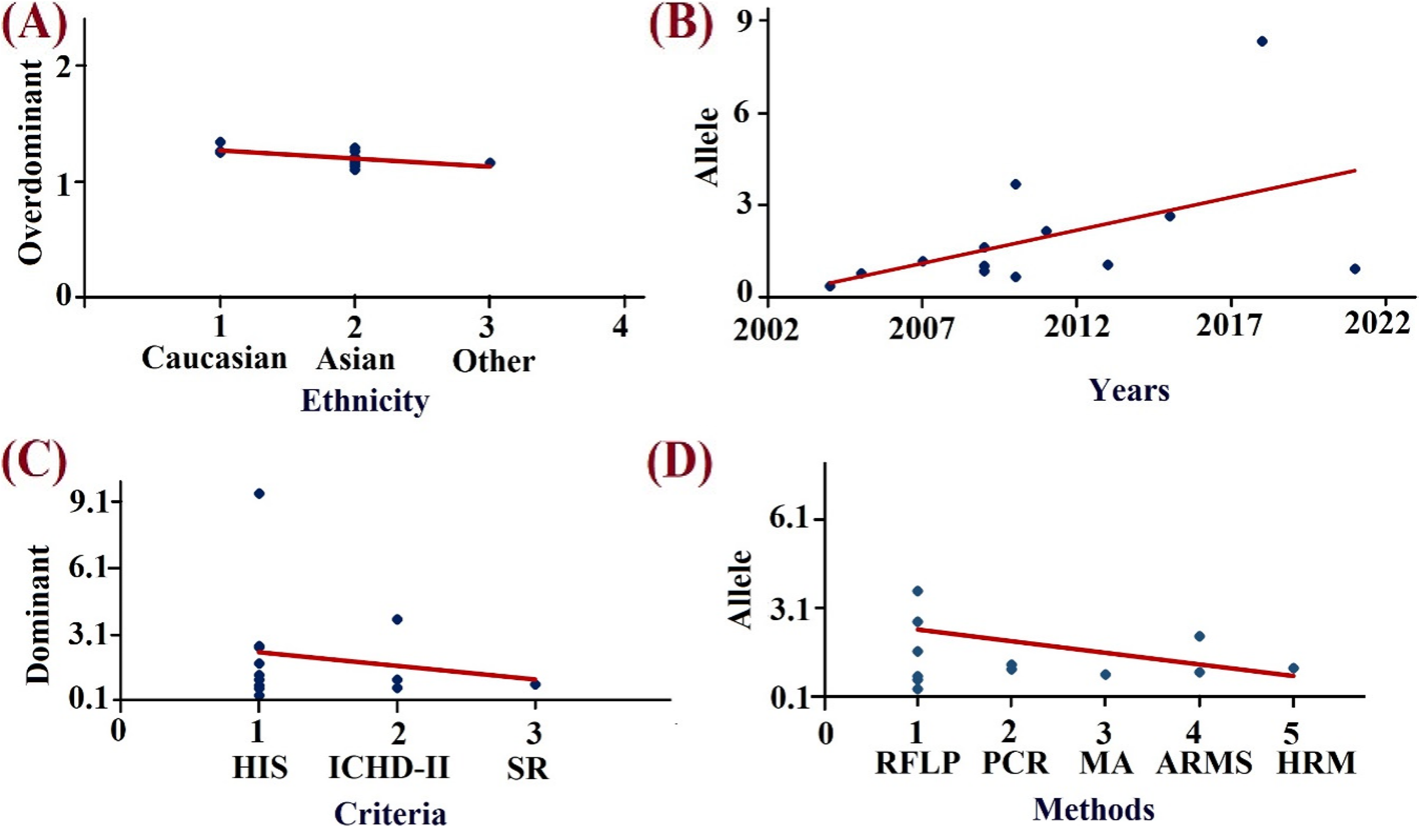
Meta regression **A** Regression Analysis Plot (RAP) showing the ethnicity as an independent variable for over dominant model’s OR. **B** RAP showing the year independent variable for allelic model’s OR. **C** RAP showing the diagnostic criteria as independent variable for the prediction of dominant model’s OR. **D** RAP showing the genotyping method as independent variable for allelic model’s OR

To identify possible causes of variation among acceptable papers based on different factors such as ethnicity (Fig. 8A), year (Fig. 8B), criteria (Fig. 8C), and genotyping technique (Fig. 8D), we used meta-regression analysis. The meta-regression analysis showed that the majority of the predicted heterogeneity parameters were not responsible for such heterogeneity (Table 6) in contrast to the ethnicity in the over-dominant genetic model where a significantly (*p* value = 0.02) strong linear relationship was observed (*R* = 0.6184) (Table 6).

**Table 6:**
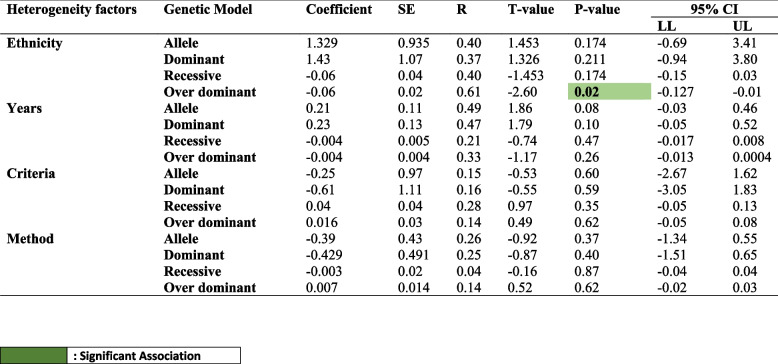
Meta-regression analyses of a potential source of heterogeneity

## Discussion

TNF-alpha is a pro-inflammatory cytokine that appears to play a role in migraine pathogenesis by simulating Calcitonin gene-related peptide (CGRP) [41]. The expression of the protein has been found to be regulated by the presence of common functional SNP i.e., -308 G/A both in vivo and in vitro [12], and is responsible for the altered levels [13] such as in Cerebrospinal fluid (CSF), plasma, and urine concentrations [37, 42–47] in migraine patients. Diverse independent research studies and meta-analyses [18–21], have found the conflicting result on the association between -308 G/A variant and the risk of migraine. Therefore, we conducted an updated meta-analysis with TSA and meta-regression to understand the relationship between -308 G > A transition and migraine susceptibility more precisely.

The present meta-analysis did not show any significant association between the rs1800629 and migraine and its clinical phenotype (MA) after utilizing different genetic models. Interestingly, after sub-grouping using the “ethnicity criteria” in the migraine group, it was found that the dominant model showed a slightly significantly higher risk of migraine than the allelic model (Table 3) in the Asian population. Also, the recessive, and over-dominant models increase the risk of the condition in Asian ethnic groups but the association did not reach statistical significance (Table 3). In addition, we didn’t observe any significant association in the Caucasian population in contrast to the Egyptian population which shows a considerably higher risk of migraine utilizing different genetic models (Table 3).

Comparing our meta-analytic data with the pre-existing meta-analysis, the results were found consistent [18–21] in contrast to Chen and group [20]. This difference might be due to the inclusion of studies [27–30, 34, 35] belonging to the Asian population only by Chen and group (Chen et al., 2015). Concerning the risk association between MA and -308 A variant, the present study didn’t find any significant association after utilizing different genetics models which were not consistent with the pre-existing meta-analysis results [18–20]. Interestingly, it is important to note that the risk variant showed a strong association in females regarding both migraines as well in MA phenotype [18, 20], which was not observed in the present study. Apart from such conflicting and diverse results, all meta-analyses confirmed that the risk of migraine was greater in the Asian population than in the Caucasian population concerning the risk allele of TNF alpha (rs1800629). In addition, we also observe a significant association in the Egyptian population which shows a considerably higher risk of migraine utilizing a different genetic model. But this large effect might be due to the presence of a single study and might be changed if more studies from Egyptian ethnicity will be included.

There are many reasons why a lack of association occurs and the prime reason might be the limited number of studies in the meta-analysis as shown (Fig. 7), also there might be due high degree of heterogeneity among the studies investigating, inherent biases such as publication, sampling, and selection bias (only English published literature) can’t be avoided in a meta-analysis of observational studies. The disparity between the different meta-analysis studies which we observe in the present study related to the risk of MA in presence of -308A rare variant might result from diverse reasons which might be the varied sample size, population stratification, utilization of the diagnostic criteria, and also the criteria utilized for the selection of research studies for meta-analysis. Varied sample sizes could impact the association and frequency and one such example is the cohort study [32] which was much more powered than the other studies. But, after using sensitivity analysis, there was no such indication for the dominance of a large study on the pooled result. According to population stratification theory, systematic differences in the frequency of alleles between sub-populations are caused by several processes including distinct ancestry, non-random mating, and geographic isolation [48]. Utilization of the strict or modified IHS classification criteria and updated ICHD-3, there is a chance for the misclassification of migraine types due to its biological heterogeneity and wide clinical spectrum [49]. But using a “Bayesian meta-regression” analysis, the present study did not find any significant cause of heterogeneity which mighty be the factor for the heterogeneity (Table 6).

Regarding the strength of the present meta-analysis from the previous meta-analysis [18–21] first, this meta-study included a large number of subjects (cases: 7193 and controls: 23,091) and featured with the TSA and meta-regression. Second, the NOS quality scale was utilized for the inclusion of studies based on their quality. Thirdly and notably, only the studies were included that were found to be in the HWE, and this resulted in the inclusion of only 13 studies after 18 studies were selected (Table 2). Additionally, we have also incorporated the meta-regression to find out the variable responsible for the heterogeneity, which was not previously mentioned in any meta-analysis of TNF alpha -308 G > A and migraine risk. Apart from the strengths, a major limitation of our meta-analysis was that we did not observe any relationship between migraine and -308G/A utilizing the gender variable which was observed by [18, 20]. Also, we did not observe any gene–gene or gene-environmental interaction and risk of migraine. Because of the possibility of population stratification and the small number of studies (Fig. 7), the results of these subgroup analyses should be interpreted with caution. Enclosing the section, interestingly, all meta-analysis to date including the present study supports the fact that the rare variant i.e., -308A increases the risk of migraine significantly in the Asian ethnic group. Therefore, this study might explain the effect of risk variation on migraine susceptibility based on ethnicity.

## Conclusion

In the current meta-analysis, no overall association was found between the risk variant and susceptibility to migraine and its clinical type i.e., MA but after subgrouping, the increased risk was observed between -308 G/A & migraine in Asian population [9, 24].

## Data Availability

All data generated or analyzed during this study are included in this article. Further enquiries can be directed to the corresponding author.

## Abbreviations

*TNF-α*: Tumor Necrosis Factor- Alpha
ICHD-3: International Classification of Headache Disorders-3
MA: Migraine with Aura
MO: Migraine without Aura
CM: Chronic Migraine
CNS: Central Nervous System
G > A: Guanine > Adenine
OR: Odds Ratio
CI: Confidence Interval
NOS: Newcastle–Ottawa quality assessment scale
GUI: Graphical User Interphase
MRA: Meta-Regression Analysis
TSA: Trial Sequential Analysis
PRISMA: Preferred Reporting Items for Systematics Reviews and Meta-Analysis
GABA: Gamma-Aminobutyric Acid
SNPs: Single Nucleotide Polymorphisms
RIS: Required Information Size
CSF: Cerebrospinal fluid
GCRP: Calcitonin gene-related peptide
SNV: Single Nnucleotide Variations
